# ^129^Xe and Free‐Breathing ^1^H Ventilation MRI in Patients With Cystic Fibrosis: A Dual‐Center Study

**DOI:** 10.1002/jmri.28470

**Published:** 2022-10-11

**Authors:** Helen Marshall, Andreas Voskrebenzev, Laurie J. Smith, Alberto M. Biancardi, Agilo L. Kern, Guilhem J. Collier, Piotr A. Wielopolski, Pierluigi Ciet, Harm A. W. M. Tiddens, Jens Vogel‐Claussen, Jim M. Wild

**Affiliations:** ^1^ POLARIS, Imaging Sciences, Department of Infection, Immunity & Cardiovascular Disease University of Sheffield Sheffield UK; ^2^ Institute for Diagnostic and Interventional Radiology Hannover Medical School Hannover Germany; ^3^ Biomedical Research in Endstage and Obstructive Lung Disease Hannover (BREATH) German Center for Lung Research (DZL) Hannover Germany; ^4^ Department of Radiology and Nuclear medicine Erasmus MC Rotterdam The Netherlands; ^5^ Department of Pediatric Pulmonology and Allergology Sophia Children's Hospital, Erasmus MC Rotterdam The Netherlands

**Keywords:** ventilation, MRI, xenon‐129, proton, cystic fibrosis

## Abstract

**Background:**

Free‐breathing ^1^H ventilation MRI shows promise but only single‐center validation has yet been performed against methods which directly image lung ventilation in patients with cystic fibrosis (CF).

**Purpose:**

To investigate the relationship between ^129^Xe and ^1^H ventilation images using data acquired at two centers.

**Study type:**

Sequence comparison.

**Population:**

Center 1; 24 patients with CF (12 female) aged 9–47 years. Center 2; 7 patients with CF (6 female) aged 13–18 years, and 6 healthy controls (6 female) aged 21–31 years. Data were acquired in different patients at each center.

**Field Strength/Sequence:**

1.5 T, 3D steady‐state free precession and 2D spoiled gradient echo.

**Assessment:**

Subjects were scanned with ^129^Xe ventilation and ^1^H free‐breathing MRI and performed pulmonary function tests. Ventilation defect percent (VDP) was calculated using linear binning and images were visually assessed by H.M., L.J.S., and G.J.C. (10, 5, and 8 years' experience).

**Statistical Tests:**

Correlations and linear regression analyses were performed between ^129^Xe VDP, ^1^H VDP, FEV_1_, and LCI. Bland–Altman analysis of ^129^Xe VDP and ^1^H VDP was carried out. Differences in metrics were assessed using one‐way ANOVA or Kruskal–Wallis tests.

**Results:**

^129^Xe VDP and ^1^H VDP correlated strongly with; each other (*r* = 0.84), FEV_1_ z‐score (^129^Xe VDP *r* = −0.83, ^1^H VDP *r* = −0.80), and LCI (^129^Xe VDP *r* = 0.91, ^1^H VDP *r* = 0.82). Bland–Altman analysis of ^129^Xe VDP and ^1^H VDP from both centers had a bias of 0.07% and limits of agreement of −16.1% and 16.2%. Linear regression relationships of VDP with FEV_1_ were not significantly different between ^129^Xe and ^1^H VDP (*P* = 0.08), while ^129^Xe VDP had a stronger relationship with LCI than ^1^H VDP.

**Data Conclusion:**

^1^H ventilation MRI shows large‐scale agreement with ^129^Xe ventilation MRI in CF patients with established lung disease but may be less sensitive to subtle ventilation changes in patients with early‐stage lung disease.

**Evidence Level:**

2

**Technical Efficacy:**

Stage 2

Hyperpolarized (HP) gas MRI using ^129^Xe or ^3^He provides ventilation images with signal directly proportional to the density of the inhaled tracer gas.^1^ From these images, the proportion of the lung that is nonventilated, known as the ventilation defect percent (VDP), can be calculated.^2^ HP gas MRI is highly sensitive to early‐stage lung disease, disease progression and antibiotic treatment in children with CF, it is also repeatable and sensitive to the effects of acute exercise.^3^, ^4^, ^5^, ^6^, ^7^ Nevertheless, HP gas MRI is not yet widely available and requires additional equipment that is costly.

Free‐breathing ^1^H MRI can produce surrogate maps of ventilation without the use of a contrast agent.^8^ ^1^H ventilation metrics correlate strongly with lung clearance index (LCI), show good short‐term reproducibility, and can detect ventilation changes following antibiotic treatment in children with cystic fibrosis (CF).^9^, ^10^, ^11^ However, only single‐center validation has been performed against methods which directly image lung ventilation in patients with CF.^11^, ^12^, ^13^

Recent work comparing ^129^Xe and free‐breathing ^1^H ventilation MRI in children with CF showed that ^129^Xe and ^1^H VDP were both significantly greater in CF patients undergoing pulmonary exacerbation than healthy controls, but no significant difference was found between clinically stable CF patients with normal forced expiratory volume in 1 second (FEV_1_) and controls.^12^ Another study from the same site found that both ^129^Xe and ^1^H VDP decreased significantly following antibiotic treatment in eight children with CF.^11^ In both studies ^1^H VDP of the single coronal slice acquired with ^1^H free‐breathing MRI correlated with ^129^Xe VDP, lung clearance index (LCI) and forced expiratory volume in 1 second (FEV_1_). Eight children with CF were also included in a larger study (including 20 patients with chronic obstructive pulmonary disease (COPD) and 6 healthy volunteers) comparing free‐breathing ^129^Xe and ^1^H ventilation MRI that found a close relationship between the imaging techniques.^13^

It is increasingly recognized that multiple‐center studies and standardization of imaging techniques and outcome metrics are essential to transition novel imaging techniques such as ^129^Xe and ^1^H ventilation MRI into tools which can be employed for international clinical research and drug development.^14^, ^15^

The aim of this work was to investigate the relationship between ^129^Xe and ^1^H ventilation images across the whole lungs of patients with a broad spectrum of CF lung disease, using data acquired at two centers.

## Methods

### Center 1: University of Sheffield, UK

The study was approved by the Yorkshire and the Humber ‐ Leeds West research ethics committee (16/YH/03391). All adult patients and parents/guardians of children provided written informed consent.

### Center 2: Hannover Medical School, Germany

The study was approved by the institutional review board of Hannover Medical School. All adults and parents/guardians of children provided written informed consent.

### Center 1

Inclusion criteria: patients with CF older than 5 years who were clinically stable for 4 weeks prior to scanning. Exclusion criteria: FEV_1_ < 30% predicted within the previous 6 months, MRI contraindication or pregnancy.

### Center 2

Patients with CF, inclusion criteria: diagnosis of CF and aged 12–60 years. Exclusion criteria: respiratory tract exacerbation within the last month, chronic oxygen therapy, any other severe comorbidities that could limit imaging, MRI contraindication or pregnancy.

Healthy controls, inclusion criteria: aged 18–60 years. Exclusion criteria: lung disease within the last month, known history of chronic lung disease, known history of congenital lung disease, MRI contraindication or pregnancy.

### Center 1

A total of 24 patients with CF were scanned using a 1.5 T wholebody MRI system (GE Signa HDx, Milwaukee, WI). Patients underwent spirometry, multiple breath washout and body plethysmography on the same day.

### Center 2

Seven patients with CF and six healthy volunteers were scanned using a 1.5 T whole‐body MRI system (Siemens Avanto, Erlangen, Germany). Patients performed spirometry and body plethysmography on the same day, healthy volunteers performed spirometry on the same day.

### ^129^Xe Ventilation MRI

#### CENTER 1

Patients were positioned supine in a ^129^Xe transmit‐receive coil (Clinical MR Solutions, Brookfield, WI). A mix (0.50–1 L) of hyperpolarized ^129^Xe (~25% polarization, 86% ^129^Xe, 0.35–0.5 L) and N_2_ was inhaled from functional residual capacity (FRC), with inhaled gas volumes determined by patient height (details in the online repository [OR] Supplementary material S1; Table 1). Ventilation images were acquired during breath‐hold directly after inhalation of the ^129^Xe and N_2_ mixture using a 3D coronal steady‐state free precession (SSFP) sequence with full lung coverage (voxel size = 3.5 × 3.5 × 10 mm to 4.5 × 4.5 × 10 mm, flip angle = 10°, echo time (TE)/repetition time (TR) = 2.2/6.7 msec, bandwidth (BW) = 16 kHz, duration = 14 seconds; Table 1).^16^

**TABLE 1:** Ventilation Sequence Parameters

	Center 1		Center 2	
Parameter (unit)	^129^Xe Ventilation	^1^H PREFUL	^129^Xe Ventilation	^1^H PREFUL
Sequence	3D SSFP	2D SPGR	3D SSFP	2D SPGR
Coverage	Full lung	Seven coronal slices	Full lung	Seven coronal slices
Slice thickness (mm)	10	15	15	15
Slice gap (mm)	0	5	0	0 or 5
Matrix	100 × 80	128 × 128	96 × 96	128 × 128
FOV (cm)	35–45	48	38.4	38–50
Flip angle (°)	10	4	10	5
TE (msec)	2.2	0.8	1.72	0.82–0.88
TR (msec)	6.7	2.5	3.57	3.0
Bandwidth (kHz)	16	167	50	192
Temporal resolution (seconds)	N/A	0.37	N/A	0.19
Acquisition time (seconds)	14	648	5.8	333

FOV = field of view; SSFP = steady‐state free precession; SPGR = spoiled gradient echo; N/A = not applicable.

^1^H anatomical images of the same imaging volume were acquired with the in‐built transmit‐receive body coil (GE, Milwaukee, WI) using a 3D spoiled gradient echo sequence (voxel size = 3.5 × 3.5 × 5 mm to 4.5 × 4.5 × 5 mm, flip angle = 5°, TE/TR = 0.6/1.9 msec, BW = 167 kHz, duration = 4 seconds).

#### CENTER 2

Subjects were positioned supine in a linearly polarized ^129^Xe birdcage transmit coil and 16‐channel receive coil (Rapid Biomedical, Rimpar, Germany). A mix (1 L) of hyperpolarized ^129^Xe (~20%–30% polarization, 87%–92% ^129^Xe, 0.45–0.9 L) and N_2_ was inhaled from FRC, with gas volumes determined by patient age and height (details in the OR of the Supplementary material S1). Ventilation images were acquired during breath‐hold directly after inhalation of the ^129^Xe and N_2_ mixture using a 3D coronal SSFP sequence with full lung coverage (voxel size = 4.0 × 4.0 × 15 mm, flip angle = 10°, TE/TR = 1.72/3.57 msec, BW = 50 kHz, duration = 5.8 seconds; Table 1). The sequence used a stack of stars trajectory with 90 spokes per *k*‐space partition and symmetric readout. Gradient delay correction was performed assuming an isotropic delay using the method described by Herrmann et al.^17^ Relative coil sensitivities were estimated from the central portion of *k*‐space and images reconstructed using the parallel imaging/compressed sensing routine in the Berkeley Advanced Reconstruction Toolbox after Fourier transformation of data along the slice direction.^18^

^1^H anatomical images of the same imaging volume were acquired with the in‐built transmit‐receive body coil (Siemens) using a 3D spoiled gradient echo sequence (voxel size = 2.6 × 2.6 × 5 mm, flip angle = 5°, TE/TR = 0.99/3 msec, BW = 106.56 kHz, duration = 16 seconds).

### Free‐Breathing ^1^H MRI

#### CENTER 1

Patients were positioned supine in an eight‐element chest array (GE) and 250 dynamic images per slice were acquired during relaxed free‐breathing using a 2D spoiled gradient echo (SPGR) sequence with seven coronal slices centered on the carina (voxel size = 3.75 × 3.75 × 15 mm with 5 mm slice gap, flip angle = 4°, TE/TR = 0.8/2.5 msec, BW = 167 kHz, temporal resolution = 0.37 seconds, duration = 648 seconds; Table 1).

#### CENTER 2

Patients were positioned supine in a six‐element chest array (Siemens Healthcare) and 250 dynamic images per slice were acquired during relaxed free‐breathing using a 2D spoiled gradient echo (SPGR) sequence with seven coronal slices centered on the carina (voxel size = 2.97 × 2.97 × 15 mm to 3.9 × 3.9 × 15 mm with 0 mm or 5 mm slice gap, flip angle = 5°, TE = 0.82–0.88 msec, TR = 3 msec, BW = 192 kHz, generalized auto‐calibrated partially parallel acquisitions (GRAPPA) R = 2, temporal resolution = 0.19 seconds, duration = 333 seconds; Table 1).^19^

### Image Analysis

Phase‐resolved functional lung (PREFUL) analysis was performed on the free‐breathing ^1^H images from both centers, as detailed by Voskrebenzev et al, and included registration, low‐pass filtering and calculation of fractional ventilation.^20^ PREFUL analysis of center 1 data was performed at center 1 using code provided by center 2. PREFUL analysis of center 2 data was performed at center 2.

All further image analysis took place at center 1. The ^1^H anatomical images of the same imaging volume as the ^129^Xe ventilation images were registered to the ^129^Xe ventilation images using a supervised approach, that selects preregistered images, within a set computed by an in‐house software written in MATLAB (Mathworks, Natick, MA), or manually registered images, using the open‐source software itksnap, when the alignment of the automatically registered images is not satisfactory (assessed by A.M.B., 5 years' experience).^21^ In order to assist segmentation, for each PREFUL image slice, a ^1^H free‐breathing image corresponding to inspiration (i.e. with low signal in the lung parenchyma to provide high contrast at the lung boundary) was chosen manually for segmentation by H.M. (10 years' experience). Registration had previously been performed on these ^1^H free‐breathing images as part of the PREFUL reconstruction and so they were intrinsically registered to the PREFUL ventilation images.^20^

VDP (regions with no ventilation) and low ventilation percent (LVP, regions with reduced ventilation) were calculated from the ^129^Xe and PREFUL ventilation images in the same manner. The co‐registered ventilation and anatomical images were segmented automatically using spatial fuzzy C‐means thresholding to produce initial lung cavity masks that were then edited manually to remove the large airways and main vessels, and correct any segmentation errors (by L.J.S., 5 years' experience for the ^129^Xe images and H.M., 10 years' experience for the PREFUL images).^22^ Large vessels were excluded where they were visible in the anatomical ^1^H images. Linear binning of the ventilation images was performed with six bins on N4 bias‐field‐corrected images scaled by the mean signal inside the lung cavity mask.^23^, ^24^, ^25^ The resulting ventilation defect region (first bin, with signal <1/3) was used to calculate VDP and the low ventilation region (second bin, with signal <2/3) was used to calculate LVP.^23^ VDP and LVP were added to generate VDP + LVP, a further metric of abnormal ventilation. Ventilation images were assessed qualitatively by H.M., L.J.S., and G.J.C. (10, 5, and 8 years' experience, respectively).

### Pulmonary Function Tests

At both centers, spirometry and body plethysmography were performed to international standards using recommended reference equations.^26^, ^27^, ^28^ At center 1 only, multiple breath washout was performed using a modified open‐circuit Innocor (Innovision, Glamsbjerg, Denmark) and 0.2% sulfur hexafluoride (SF6).^29^

### Statistical Analysis

Statistical analysis was carried out using GraphPad Prism (San Diego, CA). The normality of data was assessed using a Shapiro–Wilk normality test and data were treated appropriately. Correlations between metrics were performed, and Bland–Altman analysis was used to assess agreement between ^129^Xe and ^1^H VDP, including in subgroups of CF patients with ^129^Xe VDP < 10%, with normal FEV_1_ and with abnormal FEV_1_. A *P*‐value < 0.05 was considered significant. To test whether ^1^H ventilation imaging was noninferior to ^129^Xe ventilation imaging, equivalence tests were performed using Wilcoxon matched‐pairs signed rank tests on the ^1^H and ^129^Xe VDP of patients with CF, with the equivalence margin set to the minimum difference to be considered real based on same‐day repeatability of ^129^Xe VDP in patients with CF; 1.6%.^7^, ^30^, ^31^ Linear regression was performed between FEV_1_ and imaging metrics, and between LCI and imaging metrics. The slopes of the ^129^Xe metric relationships with PFTs and the ^1^H metric relationships with PFTs were tested for significant difference. One‐way ANOVA with Tukey tests for multiple comparisons or Kruskal–Wallis tests with Dunn's multiple comparison tests were used to assess the differences in metrics between CF patients scanned at center 1, CF patients scanned at center 2, and controls scanned at center 2. Unpaired *t*‐tests or Mann–Whitney tests were used to assess differences between patients with a large disparity in ^129^Xe and ^1^H VDP values and those without, by applying a threshold of 5% absolute difference between ^129^Xe VDP and ^1^H VDP.

## Results

A total of 24 patients with CF (aged 9–47, median 20.6 years) were scanned at center 1. Seven patients with CF (aged 13–18, median 14.8 years) and six healthy volunteers (aged 21–31, median 24.0 years) were scanned at center 2. Patient demographics, pulmonary function test, and MRI metrics are shown in Table 2. FEV_1_ z‐score, ^129^Xe VDP, ^1^H VDP, ^129^Xe VDP + LVP, and ^1^H VDP + LVP were significantly different between CF patients scanned at center 1 and controls but not between CF patients scanned at center 2 and controls (*P* = 0.08, *P* = 0.37, *P* = 0.21, *P* = 0.23, and *P* = 0.05, respectively). ^129^Xe LVP and ^1^H LVP were significantly greater for CF patients scanned at center 2 than controls. ^129^Xe LVP was significantly lower for patients scanned at center 1 than patients scanned at center 2.

**TABLE 2:** Patient Demographics, Pulmonary Function Test and Ventilation MRI Metrics

Measure (units)	CF Patients, Center 1	CF Patients, Center 2	Controls, Center 2
n subjects (*n* female)	24 (12)	7 (6)	6 (6)
Age (years)	23.3 + 10.2	15.4 + 2.0	25.8 + 4.4
Height (cm)	163.7 + 12.7	161.1 + 6.4	170.2 + 8.6
FEV_1_ z‐score	−2.29 + 1.91^a^	−2.26 + 1.42	−0.13 + 0.88^a^
RV/TLC (%)	32.7 (17.8, 57.6)	31.5 + 10.2	‐
LCI	8.79 (6.57, 22.2)	‐	‐
^129^Xe VDP (%)	9.9 (1.1, 44.5)^a^	2.8 + 3.4	0.005 (0, 0.09)^a^
^1^H VDP (%)	13.6 + 10.7^a^	10.6 + 7.6	1.6 + 0.7^a^
^129^Xe LVP (%)	12.2 + 2.3^b^	17.1 + 6.2^b^,^c^	11.8 + 2.9^c^
^1^H LVP (%)	15.3 (11.5, 24.8)	18.3 + 3.6^c^	12.2 + 3.1^c^
^129^Xe VDP + LVP (%)	25.2 (10.6, 54.6)^a^	19.9 + 8.9	11.8 + 2.9^a^
^1^H VDP + LVP (%)	29.9 + 12.5^a^	28.9 + 10.0	13.9 + 3.5^a^

^a^ Significant difference between CF patients at center 1 and controls.

^b^ Significant difference between CF patients at center 1 and CF patients at center 2.

^c^ Significant difference between CF patients at center 2 and controls.

*n* = number; FEV_1_ = forced expiratory volume in 1 second; RV = residual volume; TLC = total lung capacity; LCI = lung clearance index; VDP = ventilation defect percent; LVP = low ventilation percent.

Presented as mean ± SD for normally distributed data and median (minimum, maximum) for non‐normally distributed data.

### Comparisons Between ^129^Xe VDP, ^1^H VDP, FEV_1_ , and LCI

**FIGURE 1:**
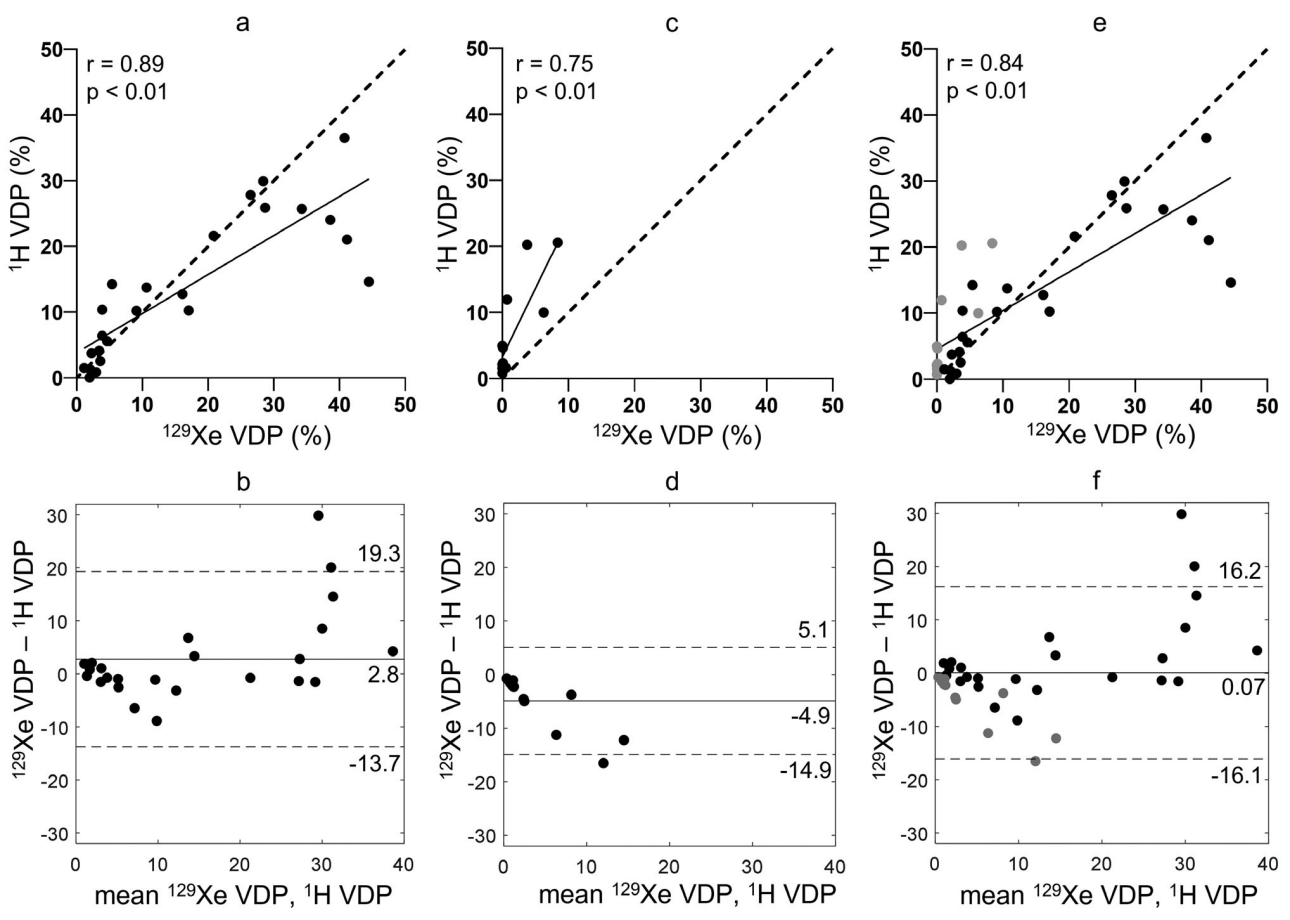
(Top row) correlation plots and (bottom row) Bland–Altman plots between ^129^Xe and ^1^H VDP for (a, b) center 1, (c, d) center 2, and (e, f) both centers (black = center 1, grey = center 2). In the correlation plots, the lines of best fit are indicated as a solid line and the lines of identity as a dashed line. In the Bland–Altman plots, bias is indicated as a solid line and limits of agreement as dashed lines.

There were significant correlations between ^129^Xe VDP and ^1^H VDP (center 1: *r* = 0.89, center 2: *r* = 0.75, and centers 1 and 2: *r* = 0.84; Fig. 1, Table 3). FEV_1_ z‐score correlated with both ^129^Xe VDP and ^1^H VDP (Table 3). LCI (measured at center 1 only) correlated with ^129^Xe VDP (*r* = 0.91) and ^1^H VDP (*r* = 0.82). There were also correlations between ^129^Xe VDP + LVP and ^1^H VDP + LVP (center 1: *r* = 0.88, center 2: *r* = 0.73, and centers 1 and 2: *r* = 0.81; Table 3; OR Fig. S3) and of ^129^Xe VDP + LVP and ^1^H VDP + LVP with FEV_1_ z‐score and LCI. The correlations of ^129^Xe LVP and ^1^H LVP with other metrics, and between themselves, were not consistent between centers (OR Table S3).

**TABLE 3:** Correlations of ^129^Xe VDP and ^1^H VDP, ^129^Xe VDP + LVP and ^1^H VDP + LVP with FEV_1_ z‐score, LCI and Each Other

	Metric 1	Metric 2	*r*	*P*
Center 1	^129^Xe VDP	^1^H VDP	0.89	4.9 × 10^−9^
	^129^Xe VDP	FEV_1_ z‐score	−0.83	4.9 × 10^−7^
	^129^Xe VDP	LCI	0.91	5.7 × 10^−10^
	^1^H VDP	FEV_1_ z‐score	−0.78	2.0 × 10^−5^
	^1^H VDP	LCI	0.82	1.1 × 10^−6^
	^129^Xe VDP + LVP	^1^H VDP + LVP	0.88	1.8 × 10^−8^
	^129^Xe VDP + LVP	FEV_1_ z‐score	−0.90	1.7 × 10^−9^
	^129^Xe VDP + LVP	LCI	0.93	2.7 × 10^−11^
	^1^H VDP + LVP	FEV_1_ z‐score	−0.78	8.1 × 10^−6^
	^1^H VDP + LVP	LCI	0.82	9.0 × 10^−7^
Center 2	^129^Xe VDP	^1^H VDP	0.75	0.005
	^129^Xe VDP	FEV_1_ z‐score	−0.70	0.01
	^1^H VDP	FEV_1_ z‐score	−0.85	3.9 × 10^−4^
	^129^Xe VDP + LVP	^1^H VDP + LVP	0.73	0.005
	^129^Xe VDP + LVP	FEV_1_ z‐score	−0.51	0.08
	^1^H VDP + LVP	FEV_1_ z‐score	−0.78	0.002
Centers 1 and 2	^129^Xe VDP	^1^H VDP	0.84	6.3 × 10^−11^
	^129^Xe VDP	FEV_1_ z‐score	−0.75	9.8 × 10^−8^
	^1^H VDP	FEV_1_ z‐score	−0.80	2.6 × 10^−9^
	^129^Xe VDP + LVP	^1^H VDP + LVP	0.81	1.0 × 10^−9^
	^129^Xe VDP + LVP	FEV_1_ z‐score	−0.77	3.1 × 10^−8^
	^1^H VDP + LVP	FEV_1_ z‐score	−0.77	2.3 × 10^−8^

For data acquired at center 1, mean ^1^H VDP was lower than mean ^129^Xe VDP with a bias of 2.8% and limits of agreement at −13.7% and 19.3% (Fig. 1b). For data acquired at center 2, mean ^1^H VDP was higher than mean ^129^Xe VDP with a bias of −4.9% and limits of agreement at −14.9% and 5.1% (Fig. 1d). When the data from both centers were pooled the bias was 0.07% and limits of agreement were −16.1% and 16.2% (Fig. 1f). When compared to ^129^Xe VDP, ^1^H ventilation MRI tended to overestimate VDP for milder disease and underestimate VDP for more severe disease. These trends were not apparent when LVP and VDP were combined (OR Fig. S3). Bland–Altman analysis between ^129^Xe VDP + LVP and ^1^H VDP + LVP showed similar magnitudes of bias and limits of agreement, with mean ^1^H VDP + LVP greater than mean ^129^Xe VDP + LVP at center 1, at center 2 and when the data from both centers were combined (OR Fig. S3).

**FIGURE 2:**
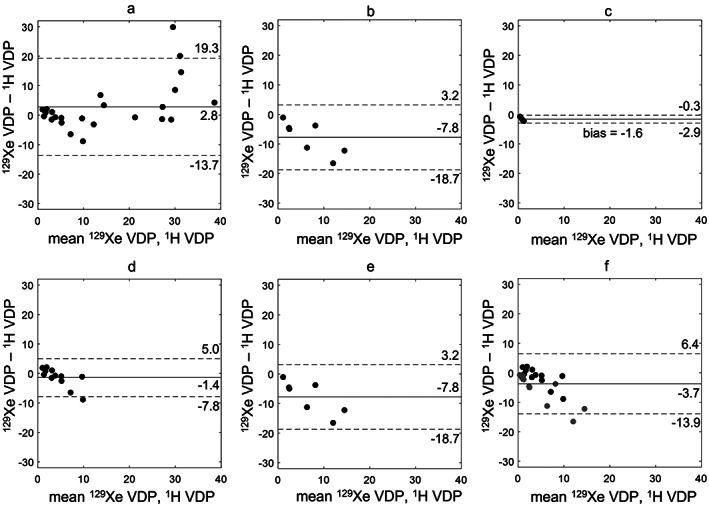
Bland–Altman plots between ^129^Xe and ^1^H VDP for (a) CF patients at center 1, (b) CF patients at center 2, (c) healthy controls at center 2, (d) CF patients at center 1 with ^129^Xe VDP < 10%, (e) CF patients at center 2 with ^129^Xe VDP < 10%, and CF patients at both centers with ^129^Xe VDP < 10% (black = center 1, grey = center 2). Bias is indicated as a solid line and limits of agreement as dashed lines.

All data acquired at center 2 had ^1^H VDP greater than ^129^Xe VDP and a trend toward increased bias for larger VDP (Fig. 1c). The bias and limits of agreement were larger for patients with CF (Fig. 2b) than healthy controls (Fig. 2c). All patients scanned at center 2 had ^129^Xe VDP < 10%. Patients scanned at center 1 with ^129^Xe VDP < 10% also showed a negative bias (Fig. 2d), although the magnitude of the bias was less than at center 2 (Fig. 2e). There were significant correlations between ^129^Xe VDP and ^1^H VDP for patients with ^129^Xe VDP < 10% (center 1: *r* = 0.73, centers 1 and 2: *r* = 0.56) (Table 4).

**TABLE 4:** Correlations of ^129^Xe VDP and ^1^H VDP for CF Patients at Each Center and With Data Pooled and for Sub‐Groups of CF Patients With ^129^Xe VDP < 10%, With Normal FEV_1_ and With Abnormal FEV_1_

	Center(s)	Age (years)	Number of Patients	*r*	*P*
All CF patients	1	20.6 (9.6, 47.5)	24	0.89	4.9 × 10^−9^
	2	14.8 (13.3, 18.4)	7	‐	‐
	1 and 2	17.7 (9.6, 47.5)	31	0.82	1.6 × 10^−8^
CF patients with ^129^Xe VDP < 10%	1	21.0 (9.6, 33.2)	12	0.73	0.007
	2	14.8 (13.3, 18.4)	7	‐	‐
	1 and 2	17.1 (9.6, 33.2)	19	0.56	0.012
CF patients with normal FEV_1_	1	21.0 (9.6, 33.2)	12	0.73	0.007
	2	17.3 (14.8, 18.4)	3	‐	‐
	1 and 2	18.2 (9.6, 33.2)	15	0.72	0.003
CF patients with abnormal FEV_1_	1	20.4 (12.0, 47.5)	12	0.49	0.10
	2	13.9 (13.3, 16.2)	4	‐	‐
	1 and 2	17.0 (12.0, 47.5)	16	0.61	0.013

Dash indicates that correlation was not performed due to low patient numbers. Age shown as median (range).

At center 1, the patients with ^129^Xe VDP < 10% were the same as the patients with normal FEV_1_. Bland–Altman analysis of patients with normal FEV_1_ showed a negative bias (OR Fig. S2a–c) and there were significant correlations between ^129^Xe VDP and ^1^H VDP (center 1: *r* = 0.73, centers: 1 and 2 *r* = 0.72) (Table 4). Patients with abnormal FEV_1_ from center 1 (who all had ^129^Xe VDP > 10%) showed a bias of 6.9% (Supplementary OR Fig. S2d) while patients with abnormal FEV_1_ from center 2 (who all had ^129^Xe VDP < 10%) showed a bias of −11.3% (Supplementary OR Fig. S2e). Pooled data showed larger limits of agreement for patients with abnormal FEV_1_ (−21.1%, 25.9%) (Supplementary OR Fig. S2f) than patients with normal FEV_1_ (−7.8, 4.3) (Supplementary OR Fig. S2c). There were significant correlations between ^129^Xe VDP and ^1^H VDP for patients with abnormal FEV_1_ when data from both centers were pooled (*r* = 0.61) but not for patients with abnormal FEV_1_ scanned at center 1 alone (*r* = 0.49, *P* = 0.1) (Table 4).

The 90% confidence intervals of the equivalence test between ^1^H and ^129^Xe VDP using all data from patients with CF were −3.1% and 2.3%. As these values fell outside the equivalence margins (−1.6%, 1.6%), equivalence between ^1^H and ^129^Xe VDP could not be established.^30^ The 90% confidence intervals of the equivalence tests also fell outside the equivalence margins (−1.6%, 1.6%) for CF patients with VDP < 10% (1.7%, 5.8%), normal FEV_1_ (0.3%, 3.1%) and abnormal FEV_1_ (−7.7, 2.9).

There were nine patients with absolute difference between ^129^Xe VDP and ^1^H VDP of more than 5%. These patients had significantly larger ^1^H VDP, worse FEV_1_ and were shorter than the other patients. ^129^Xe VDP (*P* = 0.13), age (*P* = 0.08), and RV/TLC (*P* = 0.24) were not significantly different between the groups (Supplementary OR Table S4).

**FIGURE 3:**
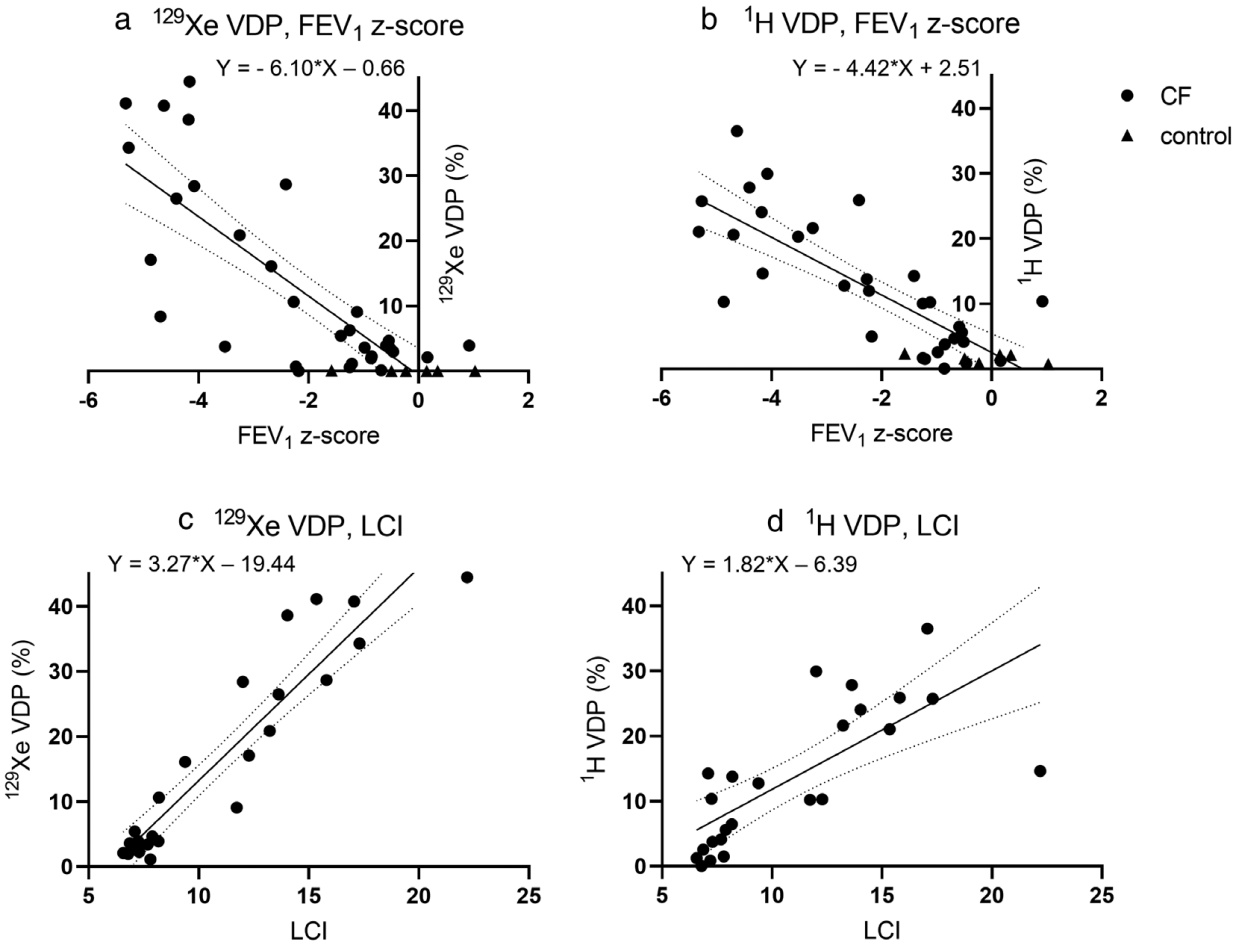
Linear regression results; (a) ^129^Xe VDP with FEV_1_ z‐score, (b) ^1^H VDP with FEV_1_ z‐score, (c) ^129^Xe VDP with LCI, and (d) ^1^H VDP with LCI. All slopes were significantly greater than zero (*P* < 0.0001).

The relationships of VDP with FEV_1_ and LCI are shown in Fig. 3. The slopes of ^129^Xe VDP with FEV_1_ (−6.10) and ^1^H VDP with FEV_1_ (−4.42) were not significantly different (*P* = 0.08). The slope of ^129^Xe VDP with LCI (3.27) was significantly greater than the slope of ^1^H VDP with LCI (1.82). The slopes of VDP + LVP with FEV_1_ and VDP + LVP with LCI were not significantly different for ^129^Xe and ^1^H (*P* = 0.52 and *P* = 0.14, respectively, Supplementary OR Fig. S4 and OR Table S5). While ^1^H LVP had significantly stronger relationships with FEV_1_ and LCI than ^129^Xe LVP had with FEV_1_ and LCI (Supplementary OR Fig. S4 and OR Table S5).

### Similarities and Differences Between ^129^Xe and ^1^H Ventilation Images

**FIGURE 4:**
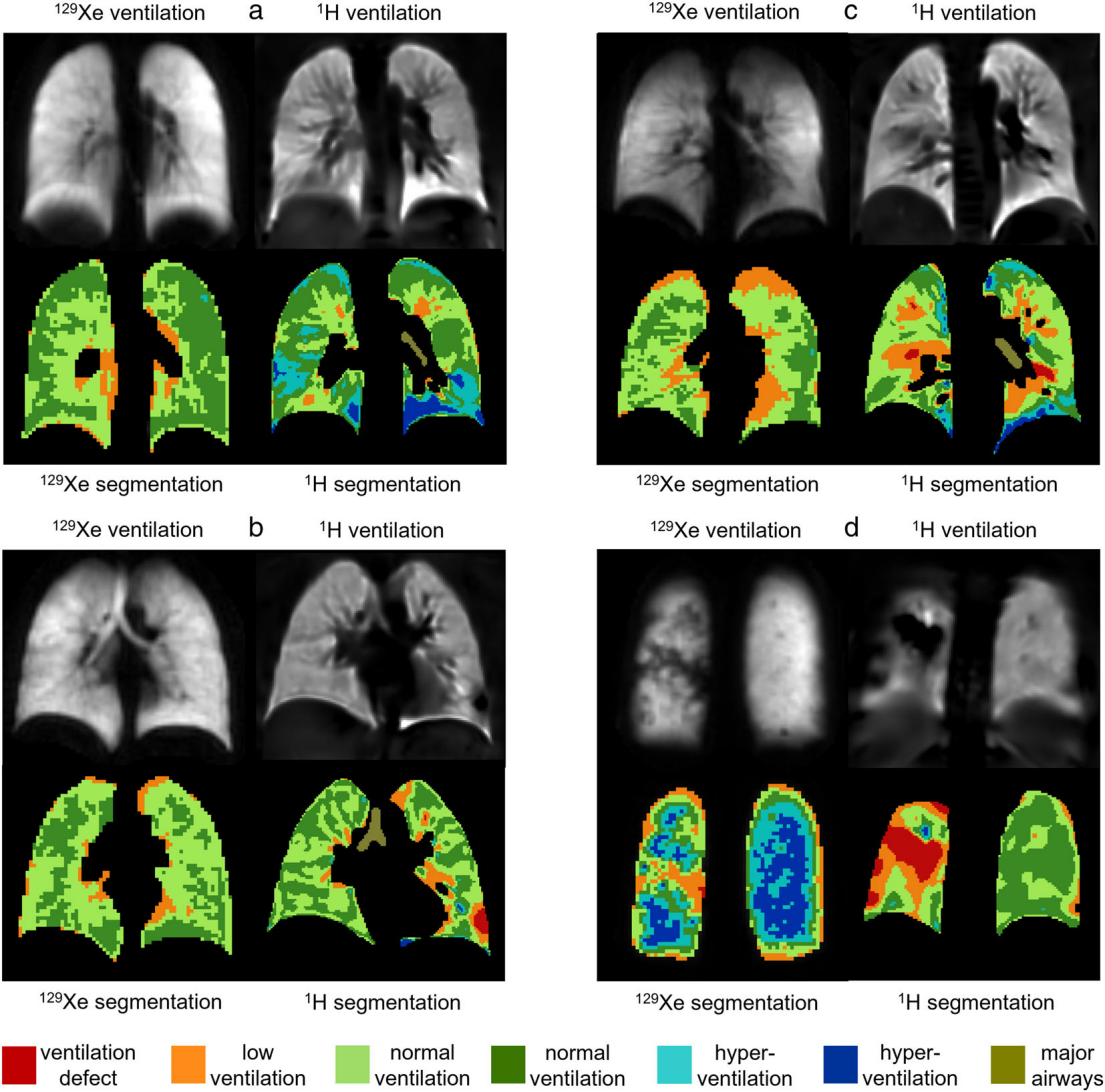
^129^Xe and ^1^H ventilation images from four controls; (a) FEV_1_ z‐score = 1.03, ^129^Xe VDP = 0.00%, ^1^H VDP = 0.7%, (b) FEV_1_ z‐score = −0.23, ^129^Xe VDP = 0.01%, ^1^H VDP = 0.9%, (c) FEV_1_ z‐score = 0.15, ^129^Xe VDP = 0.03%, ^1^H VDP = 2.1%, and (d) FEV_1_ z‐score = −1.58, ^129^Xe VDP = 0.09%, ^1^H VDP = 2.4%. Ventilation binning maps are shown with red = ventilation defect percent (VDP), orange = low ventilation percent (LVP), green = normal ventilation, blue = hyperventilation, brown = major airways. All images were acquired at center 2.

Ventilation images of controls were mostly uniform with more ventilation heterogeneity in ^1^H ventilation images than in ^129^Xe ventilation images (Fig. 4). In most cases, regions of ^1^H VDP in controls were adjacent to vessels (Fig. 4c) and the heart, however, some were not (Fig. 4b). In one control with FEV_1_ z‐score at the low end of normal (−1.58), both ^129^Xe and ^1^H ventilation images showed abnormalities in the posterior right lung (Fig. 4d). There were five controls where ventilation defects were seen on ^1^H ventilation images but not on ^129^Xe ventilation images.

**FIGURE 5:**
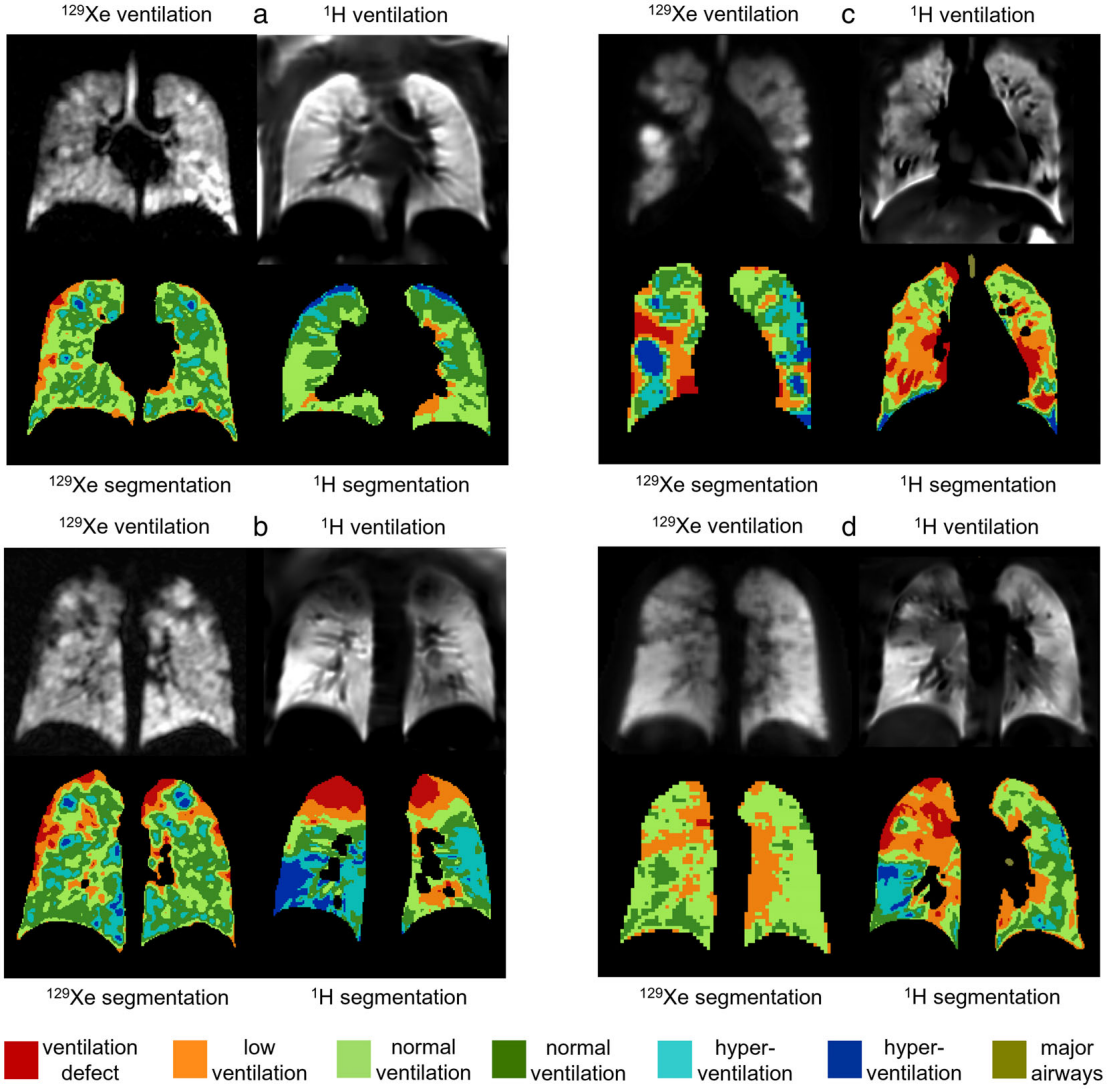
^129^Xe and ^1^H ventilation images from four CF patients with normal FEV_1_; (a) FEV_1_ z‐score = 0.16, LCI = 6.6, ^129^Xe VDP = 2.1%, ^1^H VDP = 1.3%, center 1, (b) FEV_1_ z‐score = −0.59, LCI = 8.2, ^129^Xe VDP = 3.9%, ^1^H VDP = 6.5%, center 1, (c) FEV_1_ z‐score = −1.25, ^129^Xe VDP = 6.3%, ^1^H VDP = 10.0%, center 2, and (d) FEV_1_ z‐score = −0.68, ^129^Xe VDP = 0.1%, ^1^H VDP = 4.7%, center 2. Ventilation binning maps are shown with red = ventilation defect percent (VDP), orange = low ventilation percent (LVP), green = normal ventilation, blue = hyperventilation, brown = major airways.

**FIGURE 6:**
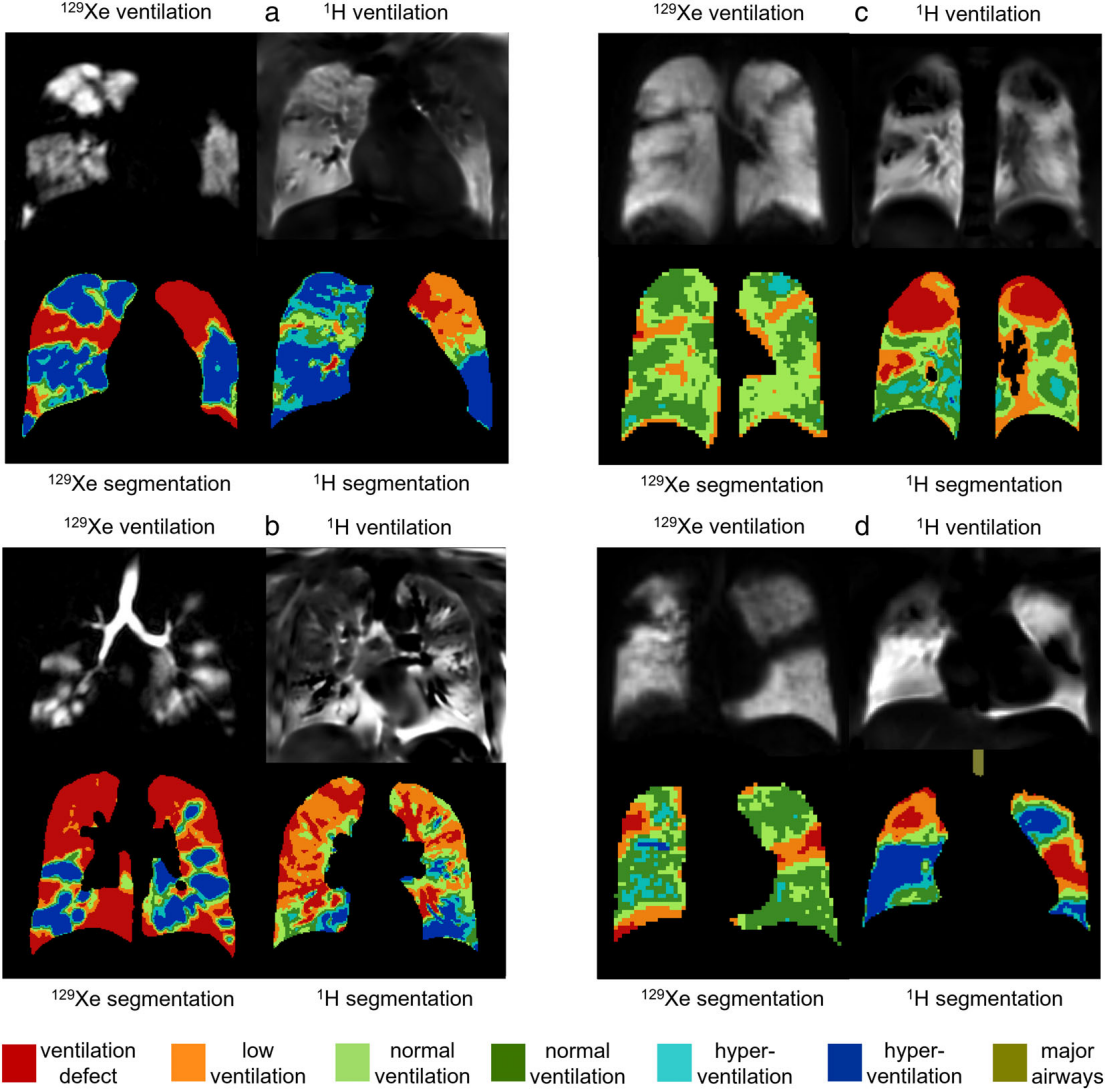
^129^Xe and ^1^H ventilation images from four CF patients with abnormal FEV_1_; (a) FEV_1_ z‐score = −4.18, LCI = 14.0, ^129^Xe VDP = 38.6%, ^1^H VDP = 24.1%, center 1, (b) FEV_1_ z‐score = −4.16, LCI = 22.2, ^129^Xe VDP = 44.5%, ^1^H VDP = 14.6%, center 1, (c) FEV_1_ z‐score = −2.23, ^129^Xe VDP = 0.7%, ^1^H VDP = 12.0%, center 2, and (d) FEV_1_ z‐score = −3.52, ^129^Xe VDP = 3.8%, ^1^H VDP = 20.3%, center 2. Ventilation binning maps are shown with red = ventilation defect percent (VDP), orange = low ventilation percent (LVP), green = normal ventilation, blue = hyperventilation, brown = major airways.

Figure 5 shows images from CF patients with normal FEV_1_ and Fig. 6 shows images from CF patients with abnormal FEV_1_. Regions of reduced ^1^H ventilation were often associated with ^129^Xe ventilation defects or heterogeneity but did not always capture their full extent or detailed patterns evident on ^129^Xe images. In general, ^1^H ventilation defects tended to be larger than ^129^Xe ventilation defects (Figs. 5b,d and 6c). There were regional similarities and differences between ^129^Xe and ^1^H ventilation images, but the distribution of medium‐to‐large scale ventilation abnormalities throughout the lungs was generally similar. There were five CF patients with normal FEV_1_ where small ventilation abnormalities (fully unventilated and partially ventilated) were observed in ^129^Xe ventilation images without corresponding regions of low or no ventilation present in ^1^H ventilation images (Fig. 5a). There was one patient with abnormal FEV_1_ where no ventilation defects were observed on ^129^Xe ventilation images and minimal ventilation defects were seen on ^1^H images.

## Discussion

Analyses of VDP and VDP + LVP show strong relationships between ^129^Xe and ^1^H ventilation images and with LCI and FEV_1_. Data were acquired at two centers from different cohorts of patients, and image analysis was standardized and performed at a single center. In linear regression analyses, relationships of VDP and VDP + LVP with FEV_1_ were not significantly different for ^129^Xe and ^1^H ventilation imaging, although ^129^Xe VDP had a significantly stronger relationship with LCI than ^1^H VDP had with LCI. There were small‐to‐moderate differences in VDP between ^129^Xe and ^1^H images for most subjects; however, there were some cases with substantially greater differences in VDP. Patients with greater differences between ^129^Xe and ^1^H VDP had higher ^1^H VDP, worse FEV_1_ and were shorter than patients with smaller differences in VDP. In patients with milder disease (VDP < 10% and normal FEV_1_) ^1^H ventilation MRI overestimated VDP, while in patients with more severe disease (abnormal FEV_1_) the relationship was less clear with underestimation of VDP at center 1 and overestimation of VDP at center 2. Qualitatively, although the appearance and exact location of ventilation defects differed between ^129^Xe and ^1^H ventilation images, larger scale ventilation patterns tended to be similar, that is, the agreement between techniques was greater for larger defects. However, statistical analysis found that ^1^H and ^129^Xe VDP could not be considered equivalent. The five cases (16% of the CF cohort) where small defects and ventilation heterogeneity present on the ^129^Xe ventilation images of CF patients with normal FEV_1_ were not detected by ^1^H ventilation imaging are suggestive that ^129^Xe ventilation imaging is more sensitive to early‐stage lung disease than ^1^H ventilation imaging. The ^1^H ventilation defects present in controls, albeit amounting to ^1^H VDPs of less than 2.5% in all controls, which tended to be adjacent to vessels, show that ^1^H ventilation imaging may have a higher susceptibility to false positive ventilation defects than ^129^Xe ventilation imaging. While medium‐sized vessels can cause reduced signal due to partial‐volume effects in ^129^Xe ventilation images, medium‐sized vessels appear to have a greater influence on ^1^H ventilation images, which can result in reduced or no signal. Regions of VDP where there was a vessel present on the matching anatomical image were removed for both ^129^Xe and ^1^H ventilation images, but sometimes ventilation defects adjacent to vessels or in the shape and potential location of vessels remained on ^1^H ventilation images contributing to VDP. Other studies have also reported ^1^H VDP in healthy volunteers despite removal of the large vessels during image analysis.^13^, ^32^ Recently, more extensive vessel segmentation using an automatic deep learning method was found to reduce ^1^H VDP and increase its reproducibility.^33^ Improved vessel segmentation such as this could be implemented to reduce this source of error for ^1^H ventilation in the future.

Differences between the ventilation imaging techniques are likely due in part to the fundamentally different sources of image contrast; inhaled ^129^Xe gas density and ^1^H signal modulation due to respiratory motion. Other factors include differing voxel size, lung coverage, lung volumes during image acquisition and some error in the matching of image planes for visual comparison. The lung volume during image acquisition was greater for ^129^Xe than ^1^H ventilation imaging (FRC plus 0.5–1 L for ^129^Xe and tidal breathing for ^1^H), and this is known to affect the appearance of ventilation defects due to airway closure/opening at lower and higher lung volumes, respectively.^34^ Most ^1^H ventilation images were acquired with a 5 mm gap between slices to reduce overall acquisition time, in contrast to the full lung coverage of ^129^Xe ventilation images, however, recently developed 3D ^1^H ventilation imaging techniques could now be applied to provide full lung coverage and increased spatial resolution with ^1^H ventilation imaging.^35^ Key differences in imaging techniques between the two centers were higher spatial resolution ^129^Xe images at center 1, a radial stack of stars *k*‐space trajectory at center 2 compared to a Cartesian *k*‐space trajectory at center 1, and the application of parallel imaging for ^1^H ventilation imaging at center 2 while auto‐calibrated parallel imaging was not available on the MRI system used at center 1. The scanners at the two centers were also made by different vendors. The thinner slices used for ^129^Xe ventilation imaging at center 1 may have contributed to higher mean ^129^Xe VDP than mean ^1^H VDP due to the reduction in partial volume effects with decreasing slice thickness, while at center 2 where both techniques used the same slice thickness mean ^129^Xe VDP was lower than mean ^1^H VDP.

The application of linear binning in the image analysis allowed user‐independent evaluation of VDP with both techniques treated equally. Partially ventilated defects often present in CF patients with milder lung disease were classified as LVP; however, the relationships of LVP with VDP, LCI and FEV_1_ were unclear particularly for ^129^Xe LVP. This is likely due to signal loss due to partial volume effects and reduced coil sensitivity also contributing to LVP. Despite this, combining LVP with VDP gave an additional metric of ventilation abnormality, which had strong correlations with LCI and FEV_1_, and showed significant differences between controls and CF patients. ^1^H ventilation MRI systematically overestimated VDP + LVP compared to ^129^Xe ventilation MRI, but there was no trend toward overestimation of VDP + LVP for milder disease and underestimation for more severe disease as was observed for VDP. VDP + LVP was not as specific as VDP, that is, the values were non‐negligible for controls due to the LVP caused by imaging effects rather than disease, but included the partially ventilated regions, which are often seen in patients with mild disease.

This dual‐center study using alike, but not identical, imaging techniques and systems at two centers, found comparable relationships between ^129^Xe and ^1^H ventilation images, showing the potential for multi‐site studies which will be necessary for these ventilation imaging techniques to be employed in large‐scale clinical research and drug development studies. Standardization of some of the imaging parameters was not possible due to the different imaging platforms at the two sites; however, image analysis was fully standardized in this study which was enabled by center 2 sharing PREFUL analysis code with center 1 and the remaining data analysis being performed by center 1.

The correlations observed between ^1^H VDP and ^129^Xe VDP, LCI and FEV_1_ were stronger than in previously published work and Bland–Altman bias and limits of agreement between ^1^H VDP and ^129^Xe VDP were smaller.^11^, ^12^ This could be due to considering the whole lungs when compared to a single slice, different acquisition parameters, field strengths and/or variations in calculating VDP between the studies. The studies also included different patient groups; clinically stable patients with CF and a broad range of disease severity in the current study (median age = 17.7 years, 55% were children), vs. children with CF undergoing pulmonary exacerbations in the study by Couch et al and Munidasa et al and clinically stable children with CF and normal FEV_1_ in Couch et al. Previous work has shown correlations of varying strength between free‐breathing ^1^H ventilation imaging and gas ventilation MRI (HP ^3^He, HP ^129^Xe and ^19^F dynamic washout) in patients with COPD, bronchiectasis, asthma and non‐small cell lung cancer.^13^, ^36^, ^37^, ^38^, ^39^

## Limitations

The CF cohorts were not well‐matched physiologically between the two sites. Despite no significant differences in age, height, FEV_1_ z‐score or RV/TLC between the two cohorts, only center 1 enrolled patients older than 18 years who would likely have more progressive, severe disease. The group of patients scanned at center 2 had a higher proportion of females than the group of patients scanned at center 1, and there were no healthy controls scanned at center 1. The small number of healthy controls and patients scanned at center 2 is also a limitation. The small sample sizes were a limitation for the statistical methods used. Differences in spatial resolution, lung coverage and lung volumes during image acquisition between ^129^Xe and ^1^H MRI, and between centers, are limitations in the context of a truly matched comparison between techniques and sites.

## Conclusions

Ventilation defect percentage calculated from ^1^H free‐breathing MRI showed strong correlations with ^129^Xe VDP, LCI and FEV_1_ in CF patients and controls scanned at two centers. When the data from both centers were pooled, bias between ^1^H VDP and ^129^Xe VDP was minimal and limits of agreement were moderately large. On a regional level, both similarities and differences between ^129^Xe and ^1^H ventilation images were observed across the range of disease severity but whole lung patterns of ventilation abnormality were similar in general. Some small defects and patchy ventilation heterogeneity observed in early‐stage CF lung disease on ^129^Xe ventilation images were not visualized with ^1^H ventilation MRI. Furthermore, imaging acquisition and analysis protocols were standardized between two centers (within MRI system capabilities). In summary, this study supports the potential use of ^1^H ventilation MRI in children and adults with CF.

## Supporting information


**Appendix S1** Supporting Information

**Figure S1** Image analysis workflow. (Top left) for ^129^Xe ventilation analysis, the best‐matching registered ^1^H anatomical images were chosen. (Bottom left) for ^1^H ventilation analysis, PREFUL analysis was performed to produce ^1^H ventilation images and a 3D ^1^H anatomical image volume was built from inspiratory images. (Right) Linear binning was performed on the ^129^Xe and PREFUL ventilation images and their matching ^1^H anatomical images in the same manner. lce = lung cavity estimation, N4 = N4 bias field correction.

**Figure S2** Bland–Altman plots between ^129^Xe and ^1^H VDP for CF patients with; (a) normal FEV_1_ at center 1, (b) normal FEV_1_ at center 2, (c) normal FEV_1_ at both centers (black = center 1, grey = center 2), (d) abnormal FEV_1_ at center 1, (e) abnormal FEV_1_ at center 2, and (f) abnormal FEV_1_ at both centers (black = center 1, grey = center 2). Bias is indicated as a solid line and limits of agreement as dashed lines.

**Figure S3** Bland–Altman plots between ^129^Xe and ^1^H VDP + LVP for (a) center 1, (b) center 2 and (c) both centers (black = center 1, grey = center 2). (d) Correlation plots between ^129^Xe and ^1^H VDP + LVP for both centers (black = center 1, grey = center 2). In the Bland–Altman plots bias is indicated as a solid line and limits of agreement as dashed lines. In the correlation plot, the line of best fit is indicated as a solid line and the line of identity as a dashed line.

**Figure S4** Linear regression results of (a) ^129^Xe LVP, (b) ^1^H LVP, (c) ^129^Xe VDP + LVP and (d) ^1^H VDP + LVP with FEV_1_ z‐score, and (e) ^129^Xe LVP, (f) ^1^H LVP, (g) ^129^Xe VDP + LVP, and (h) ^1^H VDP + LVP with LCI.
