# Supplementary material for: 129Xe and Free‐Breathing 1H Ventilation MRI in Patients With Cystic Fibrosis: A Dual‐Center Study
Source: J Magn Reson Imaging. 2022 Oct 11;57(6):1908–21. doi: 10.1002/jmri.28470 (PMC10946578; doi:10.1002/jmri.28470)
Supplement: Supplementary file 1 — Appendix S1 Supporting Information [file JMRI-57-1908-s002.docx]

Online Repository

Methods

At center 1, inhaled gas volumes were determined by patient height for all subjects as specified in OR Table 1.

OR Table 1; determination of inhaled gas volumes by patient height at center 1

| Patient height (cm) | Total bag volume (mL) | Volume of HP ^129^Xe (mL) | Volume of N_2_ (mL) |
| --- | --- | --- | --- |
| > 160 | 1000 | 500 | 500 |
| 150 - 160 | 800 | 450 | 350 |
| 140 - 150 | 650 | 400 | 250 |
| 130 - 140 | 500 | 350 | 150 |

For children (age < 18 years) scanned at center 2, inhaled hyperpolarized (HP) ^129^Xe volume was determined by height as specified in OR Table 2, while total inhaled volume was fixed at 1000 mL.

OR Table 2; determination of inhaled gas volumes by patient height for children at center 2

| Patient height (cm) | Total bag volume (mL) | Volume of HP ^129^Xe (mL) | Volume of N_2_ (mL) |
| --- | --- | --- | --- |
| > 160 | 1000 | 500 | 500 |
| 150 - 160 | 1000 | 450 | 550 |
| 140 - 150 | 1000 | 400 | 600 |

Adult patients with CF scanned at center 2 inhaled 600 mL of HP ^129^Xe and 400 mL of N_2_. Healthy controls (who were all adults) inhaled 800-900 mL of HP ^129^Xe with total inhaled volume topped up with N_2_ to 1L.

N4 bias-field-correction from ANTs version 2.2.0.0.dev297-gf23cb was used.

Results

OR Table 3; Significant correlations of ^129^Xe LVP and ^1^H LVP

|  | **Metric 1** | **Metric 2** | **r** | **p** |
| --- | --- | --- | --- | --- |
| Center 1 | ^129^Xe LVP | LCI | - 0.43 | 0.035 |
|  | ^1^H LVP | LCI | 0.53 | 0.008 |
| Center 2 | ^129^Xe LVP | ^129^Xe VDP | 0.80 | 0.002 |
|  | ^129^Xe LVP | ^1^H LVP | 0.68 | 0.011 |
|  | ^1^H LVP | ^129^Xe VDP | 0.72 | 0.008 |
|  | ^1^H LVP | ^1^H VDP | 0.71 | 0.008 |
| Centers  1 & 2 | ^1^H LVP | ^129^Xe VDP | 0.52 | 0.001 |
|  | ^1^H LVP | ^1^H VDP | 0.51 | 0.001 |
|  | ^1^H LVP | FEV_1_ z-score | - 0.37 | 0.023 |

OR Table 4; Characteristics of patients with more than or less than 5% absolute difference between ^129^Xe VDP and ^1^H VDP. Presented as mean + standard deviation for normally distributed data and median (minimum, maximum) for non-normally distributed data.

| Metric | Patients with  \|^129^Xe VDP–^1^H VDP\| > 5% | Patients with  \|^129^Xe VDP–^1^H VDP\| < 5% | p |
| --- | --- | --- | --- |
| ^129^Xe VDP (%) | 8.4 (0.7, 44.5) | 4.3 (0.0, 40.8) | 0.126 |
| ^1^H VDP (%) | 20.3 (10.4, 25.7) | 6.0 (0.1, 36.5) | 0.023 |
| FEV_1_ z-score | -4.16 (-5.32, 0.92) | -1.23 (-4.87, 0.16) | 0.032 |
| Age (years) | 13.7 (9.6, 34.5) | 18.0 (12.0, 47.5) | 0.080 |
| Height (cm) | 151.7 + 7.8 | 167.8 + 9.4 | 0.001 |
| RV/TLC | 40.4 (22.3, 57.1) | 28.5 (17.8, 57.6) | 0.237 |
| LCI | 14.7 (7.1, 22.2) | 8.2 (6.6, 12.5) | 0.177 |

OR Table 5; Linear regression results for imaging metrics with FEV_1_ z-score and LCI.

| **imaging metric** | **PFT**  **metric** | **slope** | **intercept** | **slope non-zero p-value** | **compare slope with** | **different slopes**  **p-value** |
| --- | --- | --- | --- | --- | --- | --- |
| ^129^Xe VDP | FEV_1_ | - 6.10 | - 0.66 | **< 0.0001** | ^1^H VDP | 0.08 |
| ^1^H VDP | FEV_1_ | - 4.42 | 2.51 | **< 0.0001** | - | - |
| ^129^Xe LVP | FEV_1_ | 0.11 | 13.26 | 0.77 | ^1^H LVP | **0.04** |
| ^1^H LVP | FEV_1_ | - 0.89 | 14.35 | **0.001** | - | - |
| ^129^Xe VDP+LVP | FEV_1_ | - 5.94 | 12.65 | **< 0.0001** | ^1^H VDP+LVP | 0.52 |
| ^1^H VDP+LVP | FEV_1_ | - 5.31 | 16.87 | **< 0.0001** | - | - |
| ^129^Xe VDP | LCI | 3.27 | -19.44 | **<0.0001** | ^1^H VDP | **0.0021** |
| ^1^H VDP | LCI | 1.82 | -6.39 | **<0.0001** | - |  |
| ^129^Xe LVP | LCI | -0.22 | 14.59 | **0.048** | ^1^H LVP | **<0.0001** |
| ^1^H LVP | LCI | 0.54 | 10.42 | **0.001** | - | - |
| ^129^Xe VDP+LVP | LCI | 3.01 | -4.44 | **<0.0001** | ^1^H VDP+LVP | 0.14 |
| ^1^H VDP+LVP | LCI | 2.37 | 4.04 | **<0.0001** | - | - |

OR Figure captions

OR Figure 1; Image analysis workflow. (Top left) for ^129^Xe ventilation analysis, the best matching registered ^1^H anatomical images were chosen. (Bottom left) for ^1^H ventilation analysis, PREFUL analysis was performed to produce ^1^H ventilation images and a 3D ^1^H anatomical image volume was built from inspiratory images. (Right) Linear binning was performed on the ^129^Xe and PREFUL ventilation images and their matching ^1^H anatomical images in the same manner. lce = lung cavity estimation, N4 = N4 bias field correction.

OR Figure 2; Bland-Altman plots between ^129^Xe and ^1^H VDP for CF patients with; (a) normal FEV_1_ at center 1, (b) normal FEV_1_ at center 2, (c) normal FEV_1_ at both centers (black = center 1, grey = center 2), (d) abnormal FEV_1_ at center 1, (e) abnormal FEV_1_ at center 2, and (f) abnormal FEV_1_ at both centers (black = center 1, grey = center 2). Bias is indicated as a solid line and limits of agreement as dashed lines.

OR Figure 3; Bland-Altman plots between ^129^Xe and ^1^H VDP+LVP for (a) center 1, (b) center 2 and (c) both centers (black = center 1, grey = center 2). (d) correlation plots between ^129^Xe and ^1^H VDP+LVP for both centers (black = center 1, grey = center 2). In the Bland-Altman plots bias is indicated as a solid line and limits of agreement as dashed lines. In the correlation plot the line of best fit is indicated as a solid line and the line of identity as a dashed line.

Figure 4; Linear regression results of (a) ^129^Xe LVP, (b) ^1^H LVP, (c) ^129^Xe VDP+LVP and (d) ^1^H VDP+LVP with FEV_1_ z-score, and (e) ^129^Xe LVP, (f) ^1^H LVP, (g) ^129^Xe VDP+LVP, and (h) ^1^H VDP+LVP with LCI.
