# Supplementary figures and images for: 129Xe and Free‐Breathing 1H Ventilation MRI in Patients With Cystic Fibrosis: A Dual‐Center Study
Source: J Magn Reson Imaging. 2022 Oct 11;57(6):1908–21. doi: 10.1002/jmri.28470 (PMC10946578; doi:10.1002/jmri.28470)

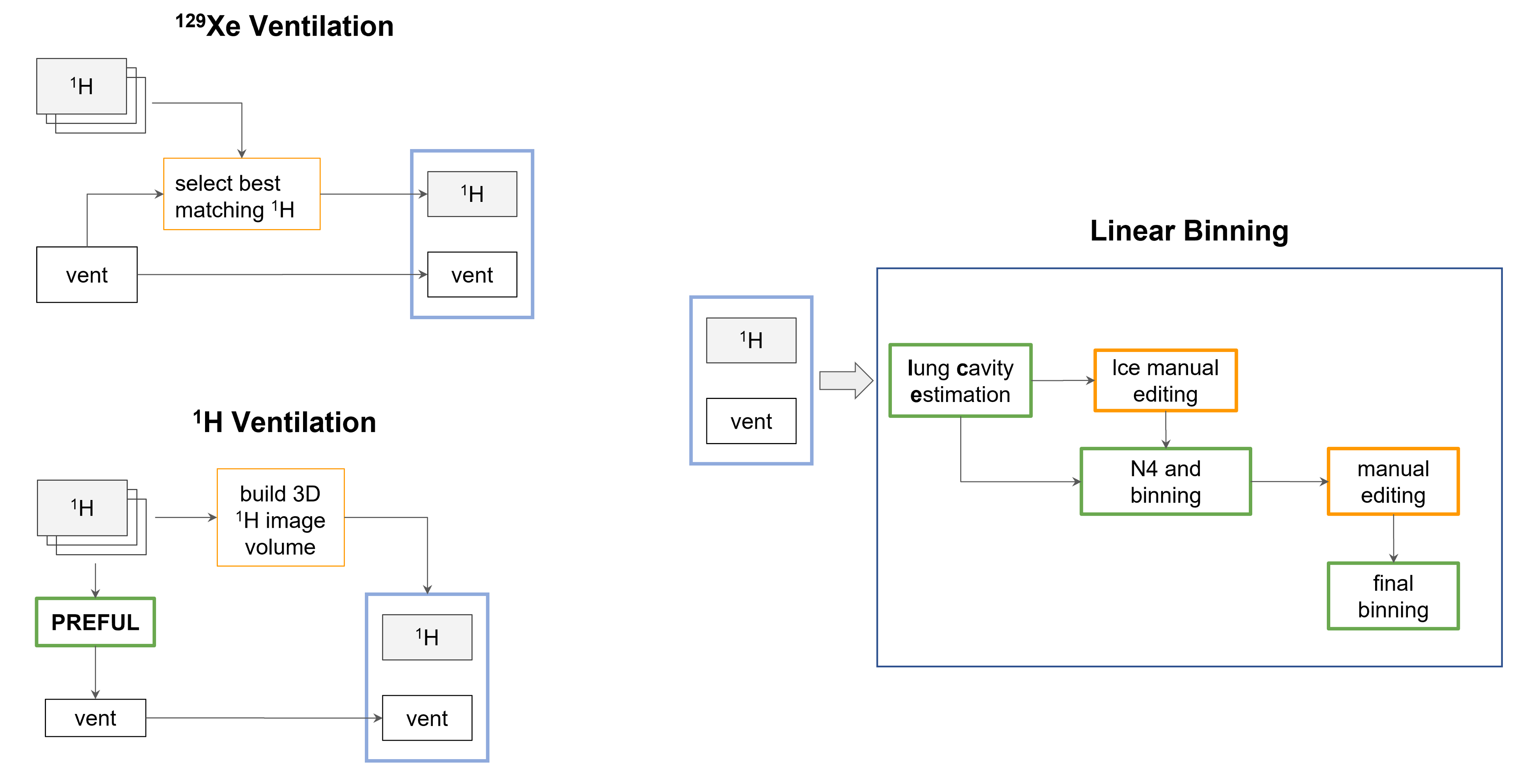

Supplement: Supplementary file 2 — Figure S1 Image analysis workflow. (Top left) for 129Xe ventilation analysis, the best‐matching registered 1H anatomical images were chosen. (Bottom left) for 1H ventilation analysis, PREFUL analysis was performed to produce 1H ventilation images and a 3D 1H anatomical image volume was built from inspiratory images. (Right) Linear binning was performed on the 129Xe and PREFUL ventilation images and their matching 1H anatomical images in the same manner. lce = lung cavity estimation, N4 = N4 bias field correction. [file JMRI-57-1908-s003.tif]

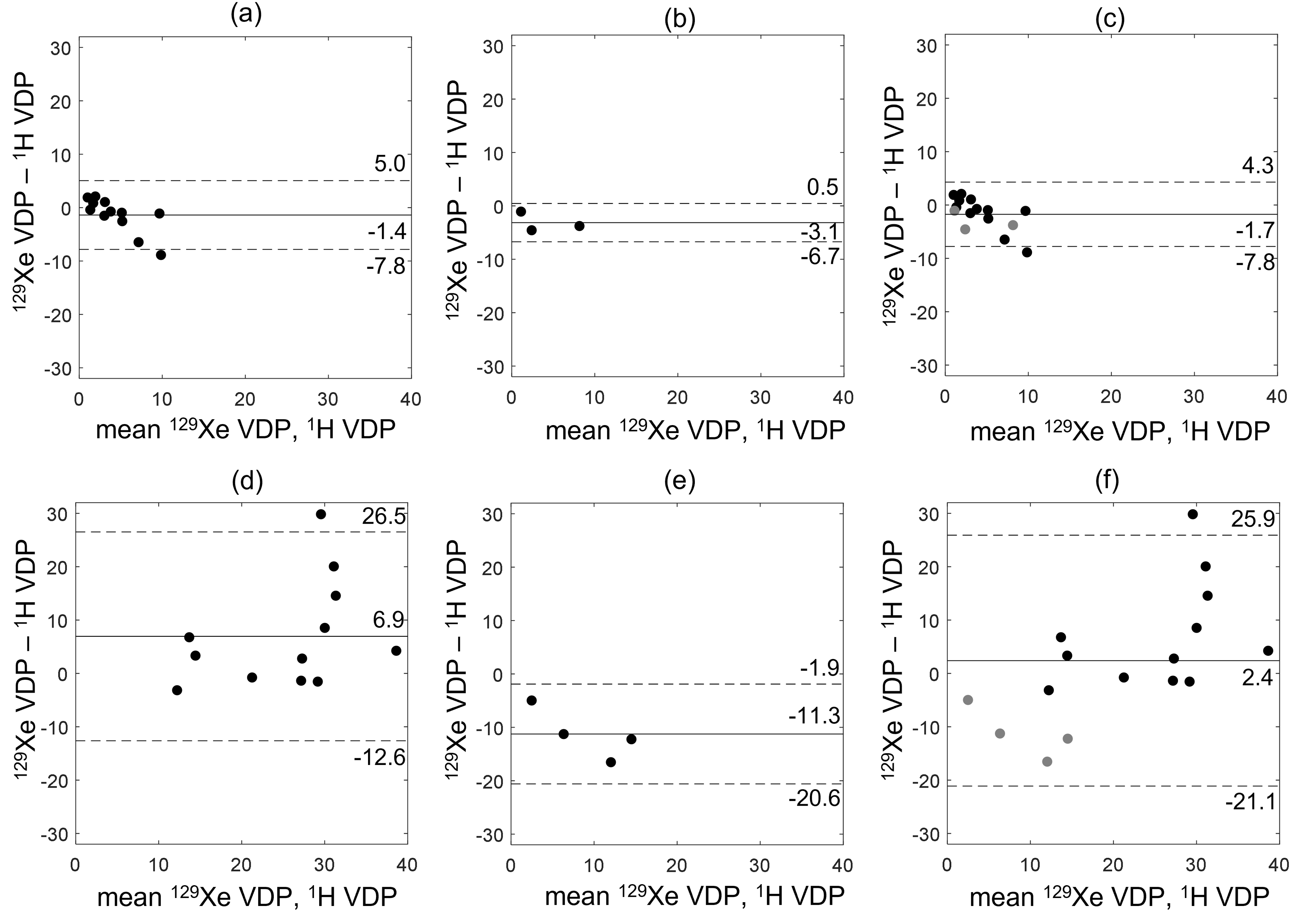

Supplement: Supplementary file 3 — Figure S2 Bland–Altman plots between 129Xe and 1H VDP for CF patients with; (a) normal FEV1 at center 1, (b) normal FEV1 at center 2, (c) normal FEV1 at both centers (black = center 1, grey = center 2), (d) abnormal FEV1 at center 1, (e) abnormal FEV1 at center 2, and (f) abnormal FEV1 at both centers (black = center 1, grey = center 2). Bias is indicated as a solid line and limits of agreement as dashed lines. [file JMRI-57-1908-s004.tif]

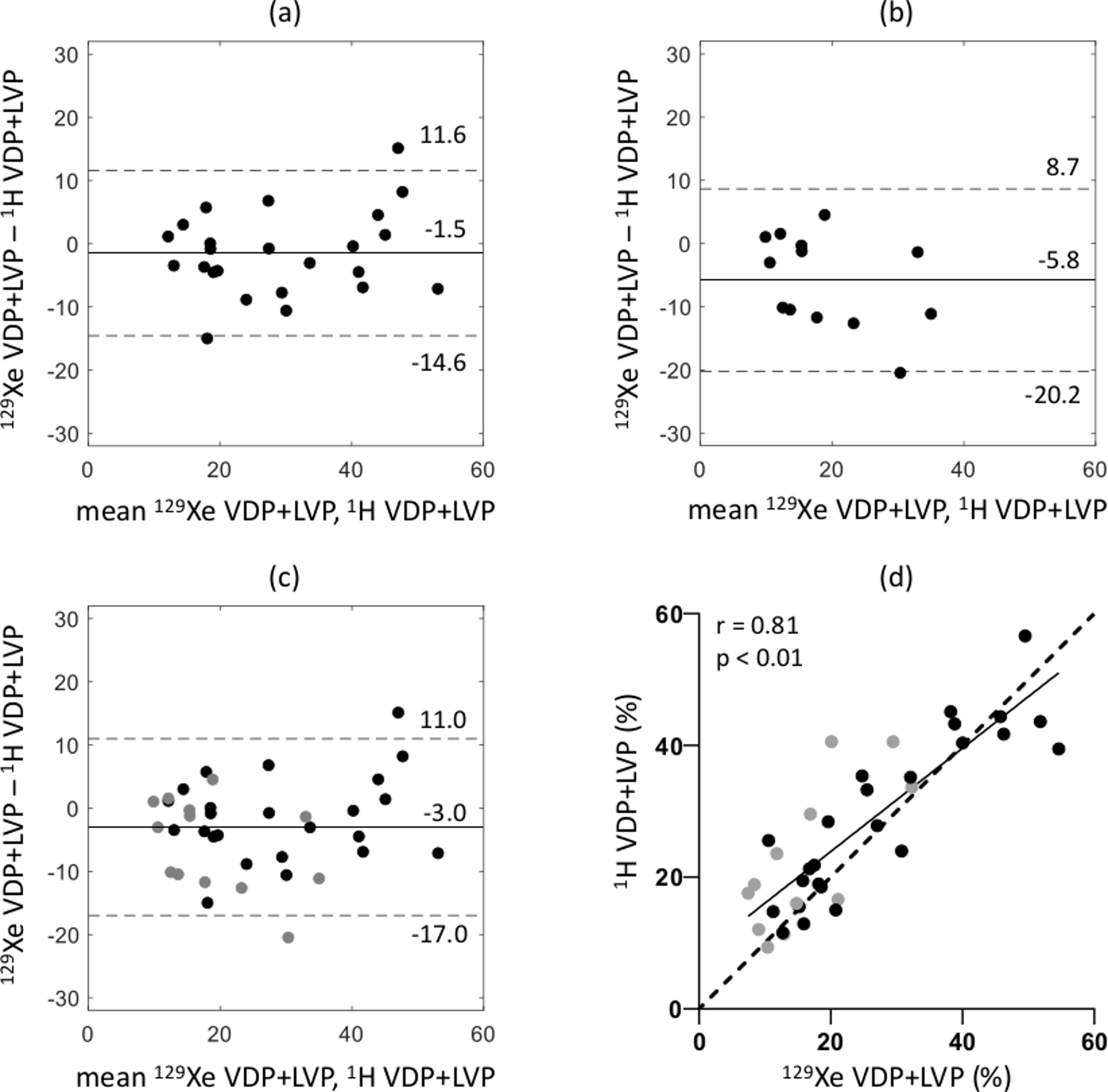

Supplement: Supplementary file 4 — Figure S3 Bland–Altman plots between 129Xe and 1H VDP + LVP for (a) center 1, (b) center 2 and (c) both centers (black = center 1, grey = center 2). (d) Correlation plots between 129Xe and 1H VDP + LVP for both centers (black = center 1, grey = center 2). In the Bland–Altman plots bias is indicated as a solid line and limits of agreement as dashed lines. In the correlation plot, the line of best fit is indicated as a solid line and the line of identity as a dashed line. [file JMRI-57-1908-s001.tif]

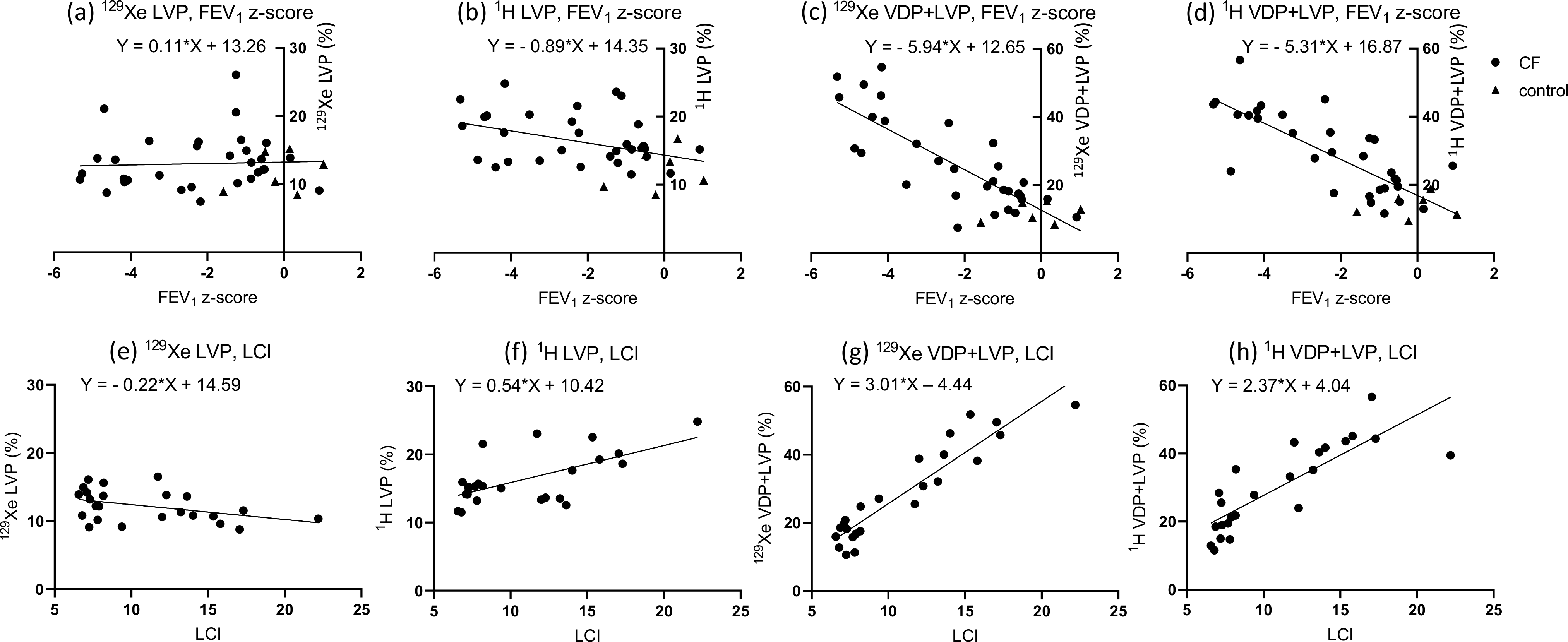

Supplement: Supplementary file 5 — Figure S4 Linear regression results of (a) 129Xe LVP, (b) 1H LVP, (c) 129Xe VDP + LVP and (d) 1H VDP + LVP with FEV1 z‐score, and (e) 129Xe LVP, (f) 1H LVP, (g) 129Xe VDP + LVP, and (h) 1H VDP + LVP with LCI. [file JMRI-57-1908-s005.tif]
